# Disseminated cysticercosis in China with complex and variable clinical manifestations: a case series

**DOI:** 10.1186/s12879-019-4171-4

**Published:** 2019-06-20

**Authors:** Yang Zou, Fei Wang, Hong-Bin Wang, Wayne W. Wu, Chia-Kwung Fan, Han-Yu Zhang, Lei Wang, Xiao-Jun Tian, Wei Li, Min-Jun Huang

**Affiliations:** 1 Beijing Institute of Tropical Medicine, Beijing Friendship Hospital, Capital Medical University; Beijing Key Laboratory for Research on Prevention and Treatment of Tropical Diseases, Beijing, China; 2 Department of Ophthalmology, Beijing Friendship Hospital, Capital Medical University, Beijing, China; 3 Department of Ophthalmology, Mayo Clinic Health System, Eau Claire, WI USA; 4 Department of Molecular Parasitology and Tropical Diseases School of Medicine College of Medicine, Taipei Medical University, Taipei, Taiwan; 5 Department of Emergency Medicine Beijing Friendship Hospital, Capital Medical University, Beijing, China

**Keywords:** Disseminated cysticercosis, Clinical manifestations, China, Case report

## Abstract

**Background:**

Cysticercosis is an emerging and neglected tropical disease (NTD) that poses a serious public health concern worldwide. Disseminated cysticercosis (DCC) is an uncommon manifestation of cysticercosis, also found in China.

**Case presentation:**

We report three cases of DCC in patients living in China, with different clinical and radiological presentations. All three patients had DCC with active ocular cysticercosis, including one patient with widespread DCC caused by direct ingestion of *Taenia solium* eggs. The intravitreal cysticercus cyst in this patient was completely extracted entirely by 23-gauge pars plana vitrectomy, and the cyst was oval in shape on the flat mount preparation.

**Conclusion:**

The clinical presentation of DCC is highly sophisticated. The diagnosis depended on the typical radiological presentations, biopsy and flat mount preparations of the cyst.

## Background

Cysticercosis is a parasitic infestation caused by the larval stage (cysticercus) of *Taenia solium*. Humans become infected by ingesting *T. solium* eggs liberated from gravid proglottids. The eggs subsequently develop into oncospheres that can hatch and penetrate the intestinal wall and circulate to the musculature. Although larvae can migrate to any human organ or tissue, the cysts can found in the central nervous system (CNS), eyes, muscles, heart, and subcutaneous soft tissues [1]. Cysticercosis is one of the most common neglected tropical diseases (NTD) in developing countries, and is endemic in most of Latin America, Sub-Saharan Africa, Southeast Asia, India, and China [2].

Disseminated cysticercosis (DCC) is an uncommon manifestation of the disease, in which the cysticerci spread to various tissues, organs or systems, such as the lesions in the CNS, eyes, muscles, and subcutaneous tissues at the same time. Although over 100 cases of DCC have been reported in the world, most of them originate from India and neighboring countries in South Asia [3–5].

In the current study, we reported three DCC cases that were diagnosed and treated in Beijing Friendship Hospital in China. In addition, we have compared these cases to others that have been reported in the literature from Chinese journals.

## Case presentation

The clinical and radiological presentations of our patients are summarized in Table 1.

**Table 1:** Symptoms and signs in three patients with DCC

		Patient 1	Patient 2	Patient 3
Area		Beijing	Henan	Helongjiang
Sex		female	male	male
Age		18	24	42
Eye	Presentation	floaters and blurred vision of left eye	diminution of vision of left eye	diminution of vision of right eye
	Intraocular pressures	normal	no test	no test
	Slip lamp examination	inflammation of left eye	no report	no report
	Fundus examination	left vitreous cyst	left extraocular cyst	right orbital cyst
		left optic disc edema	negative	negative
		vitreous cyst flat mount identified	cyst biopsy identified	cyst biopsy identified
CNS	Presentation	headache	headache	seizure
	MRI	parenchymal brain	parenchymal brain	parenchymal brain
		cerebellum	negative	negative
Soft tissues	MRI	rectus muscles	orbital tissues	orbital tissues
		facial and the neck muscles	negative	negative
		tongue	negative	negative
		diaphragm	negative	negative
		abdominal muscles	negative	negative
	X-ray	negative	forearm	chest muscles
IgG antibody	Serum	positive	positive	positive
	CSF	positive	positive	positive

*CSF* cerebrospinal fluid, *MRI* magnetic resonance imaging

### Case 1

**Fig. 1:**
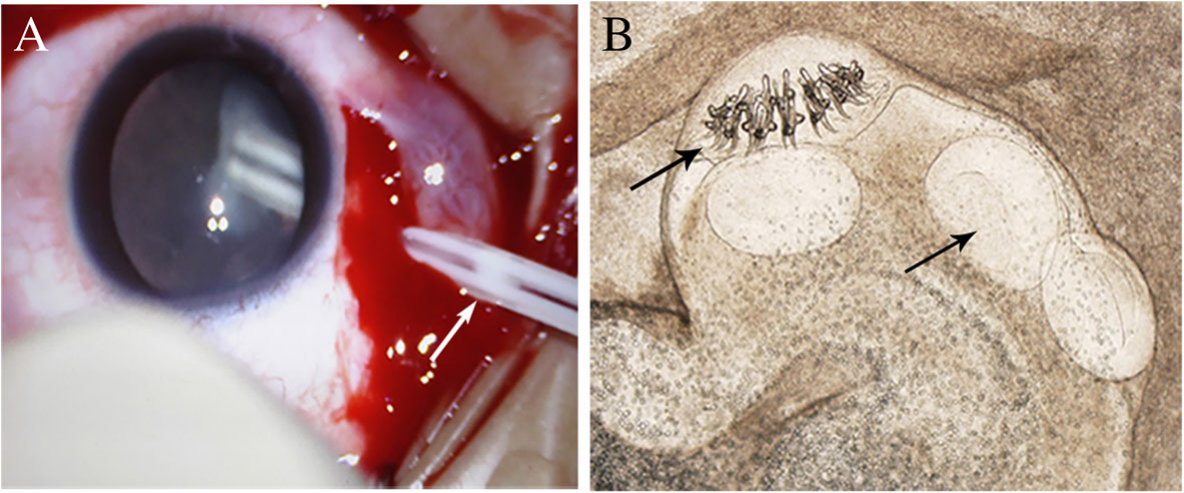
Fundus photography of the ocular cysticercus during PPV and the flat mount of the cysticercosis cyst from the eye. **a** The intact cysticercus was suctioned out with a 23G-PPV and tube. **b** The cyst contained visible scolex with its suckers and hooks

An 18-year-old Chinese female student presented at our hospital with a 1-day history of fever (temperature 39.5 °C), severe stomachache and headaches, and gradually diminished vision in the left eye. She had a history of intermittent headaches for 5 months and experienced blurred vision with shadows and floaters on the left eye for 10 days in 2016. Clinical examination in the left eye showed that the best corrected visual acuity (BCVA) was 20/40 (Snellen chart). Ultrasound imaging revealed the presence of a cyst in the vitreous cavity on the left eye. Fundus examination showed a free-floating and semi-translucent vitreous cyst in the mid-vitreous cavity and retinal vasculitis with optic disc edema. The patient underwent a 23-gauge pars plana vitrectomy (Constellation System, Alcon). The cyst was extracted in whole (Fig. 1a) with laser photocoagulation. The flat mount of the cyst was identified as oval cysticercus with a length of 0.5 cm, containing invaginated a scolex with hooklets (Fig. 1b).

**Fig. 2:**
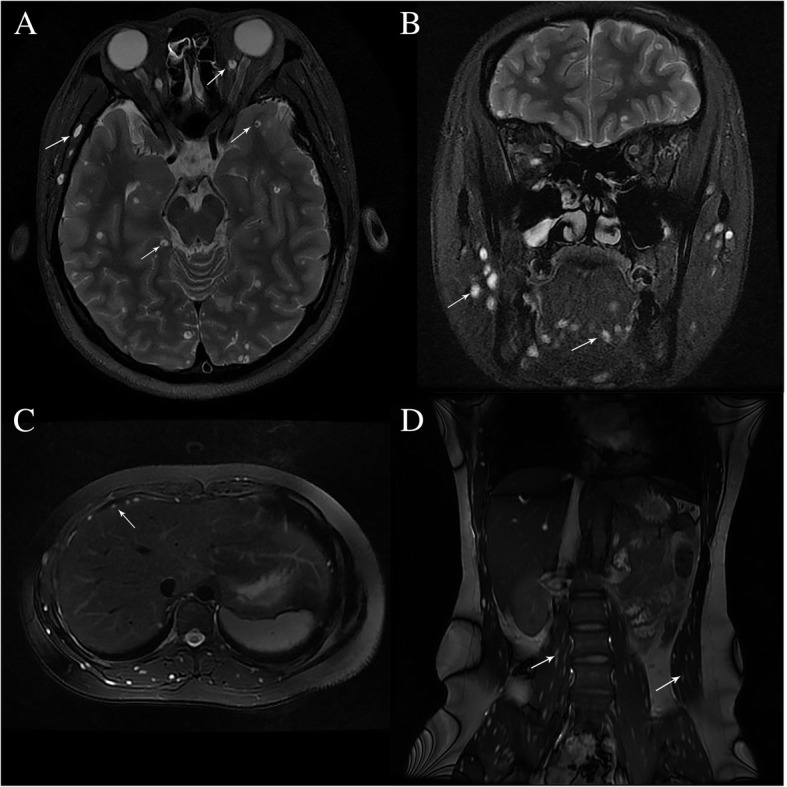
MRI of the brain, orbit, and body. Fat-suppressed T2-weighted axial and coronal images of brain and abdomen showed numerous well-defined hyperintense lesions with eccentric scolex involving the (**a**) left temporal lobe, left orbital muscle, right diaphragm, right occipital lobe, (**b**) masseter, tongue muscles, (**c**) erector spinae, and (**d**) psoas magnus muscles

Magnetic resonance imaging (MRI) with Gd-DTPA as the contrast agent was performed. MRI of the orbits and brain showed well-defined ring-enhancing cystic lesions with eccentric scolexes in the extraocular muscles, brain parenchyma (Fig. 2a), tongue, face, neck muscles, and cutaneous tissues (Fig. 2b). Abdominal MRI revealed round hyperintense lesions in the diaphragm, abdominal muscles, erector spinae, and psoas magnus muscles in T2-weighted images (T2WI) with an identifiable hypointense nidus in T1-weighted images (T1WI) and T2WI. Cystic lesions were found to be scattered around muscle tissues of the whole body (Fig. 2c and d).

Anti-*T. solium* cysticercal IgG antibodies were detected in the serum and cerebrospinal fluid (CSF) by enzyme-linked immunosorbent assay (ELISA; JL0702193, Jianlun Biology Technology Co., Ltd., Guangzhou, P.R. China). The CSF pressure was higher than 320 mmH_2_O and biochemical, cytological, and microbiological examinations of the CSF showed 8*10^6^/L WBC, higher protein level (56 mg/dL; normal range 15–40 mg/dL), normal glucose level (2.39 mmol/L in the CSF versus 4.31 mmol/L in blood), and negative bacterial culture.

**Fig. 3:**
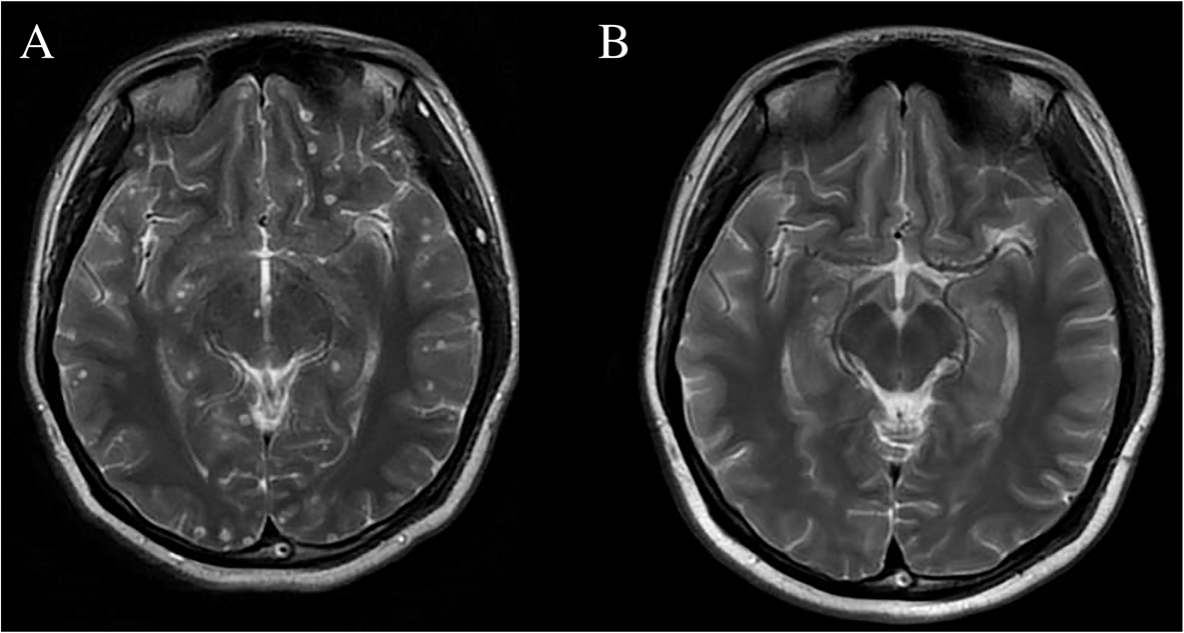
Comparison of neurocysticercosis lesions in the human brain before and after anti-parasitic treatment. **a** T2-weighted images showed numerous viable cysts before treatment. **b** A degenerating cyst in the right temporal lobe with most lesions disappeared after anti-parasitic treatment

Systemic oral albendazole and steroid treatment were initiated after the ophthalmic operation. In the first course, albendazole at a quarter of the total dose (20 mg/kg body weight daily, three times a day for 10 days) during the first course with 5 mg of intravenous dexamethasone was given daily. An acute response was observed on the third day, which was attributed to local inflammation due to the larvae death. The intravenous dose of dexamethasone was increased to 10 mg daily until albendazole treatment was discontinued. The patients received albendazole at a total dose of 20 mg/kg body weight daily for 10 days, and another two courses of albendazole equal dose with 3 months treatment interval. Most of cysts, especially intraparenchymal cysts, were disappeared (Fig. 3).

### Case 2

A 24-year-old Chinese male with no medical history was admitted to the Department of Neurology in one institution of the Henan Province with a persistent diminution of vision in the left eye, along with headaches and without seizure since 2012. The patient had been living in a rural area in the Henan Province and have never been to other provinces. He had no fever upon hospital admission. The brain MRI showed multiple left parietal cysts at the vesicular stage with edema. MRI of the orbits showed one well-defined ring-enhancing cystic lesion with eccentric scolex in the left extraocular muscle. X-ray imaging of both arms showed multiple nodular calcifications.

The detection of anticysticercal IgG antibodies by ELISA (JL0702193, Jianlun Biology Technology Co., LTD, Guangzhou, P.R. China) was positive in the serum and CSF. CSF biochemical, cytological, and microbiological examinations showed 5*10^7^/L WBCs, higher protein level (66 mg/dL), normal glucose level in the CSF and blood, and a negative bacterial culture.

After the successful ophthalmic surgery, systemic oral albendazole and steroid treatment were initiated. In the first course, the patient received albendazole (20 mg/kg body weight daily, three times a day for 10 days) with 5 mg of intravenous dexamethasone daily, and the patient had a serious headache on the 3rd day, which was attributed to local inflammation caused by the death of the larvae. Intravenous dexamethasone was increased to 10 mg daily until the albendazole treatment was discontinued. He received two additional courses of albendazole with 3-month intervals. After three treatment cycles, most of the neural cysts were effectively destroyed, and some were calcified.

### Case 3

A 42-year-old Chinese male farmer with no medical history was admitted to the Department of Neurology of one institution in the Heilongjiang Province with a possible tonic-clonic seizure witnessed by his family, along with reduction of vision in the right eye since 2015. He had been living in a rural area in Heilongjiang Province for 10 years without traveling to other areas. White tapeworm segments were found in his feces. The brain MRI showed multiple parietal cysts at the vesicular stage with edema in the frontal, parietal, and occipital white matter.

MRI of the orbits showed one well-defined ring-enhancing cystic lesion involved the right superior rectus. X-ray imaging of the chest showed multiple nodular calcifications. The detection of anticysticercal IgG antibodies by ELISA (JL0702193, Jianlun Biology Technology Co., LTD, Guangzhou, P.R. China) was positive in the serum and CSF. CSF biochemical, cytological, and microbiological examinations showed normal WBCs, normal protein level (23.52 mg/dL), normal glucose level in the CSF and blood, and a negative bacterial culture.

After the successful ophthalmic surgery, systemic oral albendazole and steroid treatment were initiated. In the first course, the patient received albendazole (20 mg/kg body weight daily, three times a day for 10 days) with 5 mg of intravenous dexamethasone, along with an oral antiepileptic drug. The patient had severe epilepsy on the 3rd day, and intravenous dexamethasone was increased to 10 mg daily. At the same time, antiepileptic therapy was given until albendazole treatment was discontinued. The patient received three additional courses of albendazole with 3-month intervals. After four treatment cycles, most of the neural cysts were effectively destroyed.

## Discussion and conclusions

The three DCC cases were all native cysticercosis in three different regions of China, including Beijing, Henan and Heilongjiang provinces. According to the data from the second report on the national survey of important human parasitic diseases in China (2004) [6], the prevalence of cysticercosis has increased from 0.011% in 1992 to 0.58% in 2004. The seroprevalence of cysticercosis was investigated in all provinces/areas of China and was detected in 31, except Beijing, Tianjin, Chongqing, Jiangsu, Zhejiang and Hunan Provinces, and. In this report, DCC cases from Beijing and Henan Province have been reported for the first time.

According to the literature, 13 DCC cases were reported from eight provinces in China from 1982 to 2011 (Table 2). These cases were from the Shanghai, Fujian, Guangxi, Sichuan, Hebei, Heilongjiang, Shandong and Yunnan Provinces of China [7–16]. In the last 8 years, almost no DCC cases have been reported in the literature. However, we have found three cases of DCC from 2012 to 2016 in China that should be included in the current literature.

**Table 2:** Demographics and presentations of DCC in 13 previous cases from China [7–16]

Case Reported Time	Number of cases	Location of Cysticercosis				Origin of Reported Case	References
		Parenchyma	Subcutaneous	Ocular	Other organs		
1988	1	+	+	+	–	Fujian Province	[7]
1991	1	+	+	+	Lung lesion	Shanghai	[8]
1992	2	+	+	orbit muscle	–	Guangxi Province	[9]
1995	1	+	+	+	–	Sichuan Province	[10]
1996	2	+	+	+	–	Hebei Province	[11]
2002	1	+	+	–	Lung lesion	Heilongjiang Province	[12]
2003	1	+	–	–	Lung lesion, Liver lesion	Shandong Province	[13]
2004	1	+	+	+	–	Guangxi Province	[14]
2004	1	+	+	–	Lung lesion	Guangxi Province	[14]
2007	1	+	+	–	Spleen lesion	Fujian Province	[15]
2011	1	+	+	–	Lung lesion, Oral mucosa lesion	Yunnan Province	[16]

(+): positive

(−): negative

Two of our three DCC cases lived in a cysticercosis-endemic area (Henan and Heilongjiang Provinces), and one case was a tapeworm carrier. Notably, the first case was infected by direct ingestion of *T. solium* eggs for body weight control, and the laboratory tests indicated that the eggs purchased online were the ova of tapeworms rather than roundworms. Data from the second national survey showed that it is not unusual that so-called white-collar staffs, such as office workers and teachers, have a high prevalence of food-borne parasitosis like cysticercosis [6]. A previous study in China also found that among a random sample of 200 cases of cysticercosis from 2004 to 2006, almost a half came from urban areas, while most infected patients had an occupational history as a farmer [17]. For this reason, it is crucial to raise health awareness and knowledge by building a comprehensive public health outreach network in the community [18].

The clinical manifestation of cysticercosis varies between patients. DCC is the most unusual cysticercosis of all forms, as it involves the brain and can be fatal in some cases. In all of the literature from China and our reported cases [7–16], all cases (16/16, 100%) were neurocysticercosis. Among them, 15 cases were associated with subcutaneous lesions, eight cases with ocular cysticercosis, five cases with pulmonary cysticercosis, and one case each associated with hepatic, splenic, and oral mucosal lesions. Among the three DCC cases in China, Case 1 presented with a very rare form of DCC. She had an active and disseminated parenchymal neurocysticercosis, ocular cysticercosis, soft tissue, and skeletal muscles cysticercosis.

Diagnostic criteria for cysticercosis was based on objective clinical, imaging, immunologic, and epidemiologic data [18]. From the 13 DCC cases, 6 cases (separately reported in 1988, 1991, 2002, 2003, 2011) were confirmed by characteristic lesions on the neuro-imaging and positive by IgG antibody positivity in serum. The rest were confirmed by subcutaneous nodule biopsy, or the eye cysts removed were confirmed as cysticercosis combined with imaging finding and positive serologic tests [7–16]. In this article, one of the three DCC cases were diagnosed by flat mount preparation of the ocular cyst.

All DCC patients should undergo surgery prior to medical treatment to remove ocular and intraventricular cysticercosis [19], given that inflammation around the degenerating cysticerci in the eye, particularly in the setting of anti-parasitic therapy, can increase the risk of blindness. Subsequently, anti-parasitic therapy was active in treating active cysts, while reducing seizure risk and the likelihood of recurrent hydrocephalus [20]. The significant risk of anti-parasitic therapy was the worsening of neurologic symptoms due to inflammation around the degenerating cyst, particularly for patients with a large number of lesions or elevated intracranial pressure. However, anti-parasitic therapy generally reduced the number of parasites and reduced seizures in patients with seizures due to viable parenchymal cysts [20].

Previous studies have shown that conventional-dose albendazole therapy (15 mg/kg per day in two daily doses up to 800 mg/day, with food) had limited effectiveness in some cases [21], and adjunctive corticosteroids should be administered prior to and during anti-parasitic therapy [19]. Multiple high doses were considered (daily albendazole at a dose of 60 mg/kg of body weight up to 1200 mg/day in three divided doses) over a relatively long course (10 days per course). Albendazole can cross the blood-brain barrier and enter parasites for better pharmacological effects, and there are minimal adverse effects associated with the drug. The radiological outcomes of motile lesions are an important treatment response indicator. Motile lesions may be stable, chronic, or calcified in patients after treatment. Timely and effective treatment may eliminate chronic lesions in the body. Since clinical symptom may relieve after MRI manifestation, it is necessary to perform MRI follow-ups one to three times after the clinical symptoms show improvements. Our three patients were considered cured with the disappearance or calcification of the lesions, as confirmed by MRI and CT after several treatment courses.

In conclusion, cysticercosis is a severe food-borne parasitic disease, and DCC presents a threat to human health in both rural and urban areas in China. The clinical presentation of DCC is highly complex and variable, including CNS, ocular, and subcutaneous symptoms. The diagnostic criteria need to consider the epidemiologic data, as well as objective clinical findings, imaging data, immunologic studies, and biopsy information. High-dosage albendazole is an effective treatment for patients with DCC.

## Data Availability

The datasets used and/or analyzed during the current study are available from the corresponding author on reasonable request.

## Abbreviations

CNS: Central nervous system
CT: Computed tomography
DCC: Disseminated cysticercosis
MRI: Magnetic resonance imaging
NCC: Neurocysticercosis
